# Supplementary material for: Leveraging natural language processing for efficient information extraction from breast cancer pathology reports: Single-institution study
Source: PLoS One. 2025 Feb 18;20(2):e0318726. doi: 10.1371/journal.pone.0318726 (PMC12005671; doi:10.1371/journal.pone.0318726)
Supplement: S1 File — (PDF) [file pone.0318726.s001.pdf]

S1 File. Comprehensive Annotation Guideline for Breast Cancer Surgical Pathology Reports

1. Purpose and Scope

This guideline aims to establish a standardized approach for annotating breast cancer surgical pathology reports. It is designed to facilitate the development of question-and-answer algorithms for efficient information extraction from 1,215 semi-structured pathology reports.

2. Reference Standards

The annotation standards are derived from comprehensive reviews of breast cancer surgical pathology reports and extensive consultations with experienced pathologists. These standards ensure that the annotations align with current medical knowledge and practices in breast cancer pathology.

3. Development Process

The guideline was developed through an iterative process involving:

- Initial draft creation based on literature review
- Multiple rounds of discussions with pathologists
- Refinement based on pilot annotations
- Final review and approval by a panel of expert pathologists

4. Annotation Methodology

- Tool: Open-source Label Studio software for manual annotation
- Preprocessing: No specific preprocessing for negative terms to maintain original report integrity
- Primary Annotation: Conducted by a health information manager with extensive pathology background to ensure consistency
- Quality Control: Secondary annotation by an independent annotator for cross-verification and accuracy assessment
- Inter-annotator Agreement: Regular meetings to resolve discrepancies and maintain annotation consistency

5. Phenotypes for Annotation

The following 19 phenotypes have been identified as crucial for characterizing breast cancer surgical pathology reports:

1. Organ: Specify the exact organ involved (e.g., left breast, right breast)
2. Tumor Site: Precise location within the breast (e.g., upper outer quadrant, retroareolar)

3. Histologic Type: Specific classification of the tumor (e.g., invasive ductal carcinoma, lobular carcinoma)
4. Intraductal Component Status: Presence and extent of ductal carcinoma in situ (DCIS)
5. Nuclear Grade: Assessment of nuclear features (Grade 1, 2, or 3)
6. Necrosis Status: Presence or absence of tumor necrosis
7. Skin (Nipple) Invasion Status: Evidence of tumor invasion into skin or nipple
8. Lymph Nodes: Number of involved nodes and total nodes examined
9. Arteriovenous Invasion: Presence or absence of tumor cells within blood vessels
10. Lymphovascular Invasion: Presence or absence of tumor cells within lymphatic vessels
11. Tumor Border: Description of tumor margins (e.g., infiltrative, pushing)
12. Microcalcification: Presence and characteristics of microcalcifications
13. Pathologic Stage: TNM classification based on pathological findings
14. Superior Margin: Distance of tumor from the superior resection margin
15. Inferior Margin: Distance of tumor from the inferior resection margin
16. Medial Margin: Distance of tumor from the medial resection margin
17. Lateral Margin: Distance of tumor from the lateral resection margin
18. Deep Margin: Distance of tumor from the deep resection margin
19. Superficial Margin: Distance of tumor from the superficial resection margin

6. Annotation Instructions

For each phenotype:

- Carefully review the entire pathology report
- Identify relevant information using exact text matches or close synonyms
- Highlight and label the corresponding text in Label Studio
- Record 'Not Reported' if information is absent
- Note any ambiguities or uncertainties for further review

7. Quality Assurance

- Regular review of annotations by senior pathologists
- Periodic inter-annotator agreement assessments
- Continuous refinement of guidelines based on encountered challenges
- Documentation of decision rules for complex cases

8. Data Management and Confidentiality

- Ensure all patient identifiers are removed before annotation
- Store annotated data in a secure, encrypted database
- Restrict access to authorized personnel only
- Comply with all relevant data protection regulations

9. Training and Support

- Provide comprehensive training to all annotators before commencing work
- Offer ongoing support and clarification for annotators
- Conduct regular team meetings to discuss challenges and share insights

10. Version Control

- Maintain a log of all changes to the annotation guideline
- Update version number with each significant revision
- Ensure all annotators are using the most current version of the guideline

This comprehensive guideline provides a structured and detailed approach for annotating breast cancer surgical pathology reports. By following these instructions, annotators can ensure consistency, accuracy, and reliability in data extraction, ultimately contributing to improved analysis and research in breast cancer pathology.
