# Supplementary figures and images for: Leveraging natural language processing for efficient information extraction from breast cancer pathology reports: Single-institution study
Source: PLoS One. 2025 Feb 18;20(2):e0318726. doi: 10.1371/journal.pone.0318726 (PMC12005671; doi:10.1371/journal.pone.0318726)

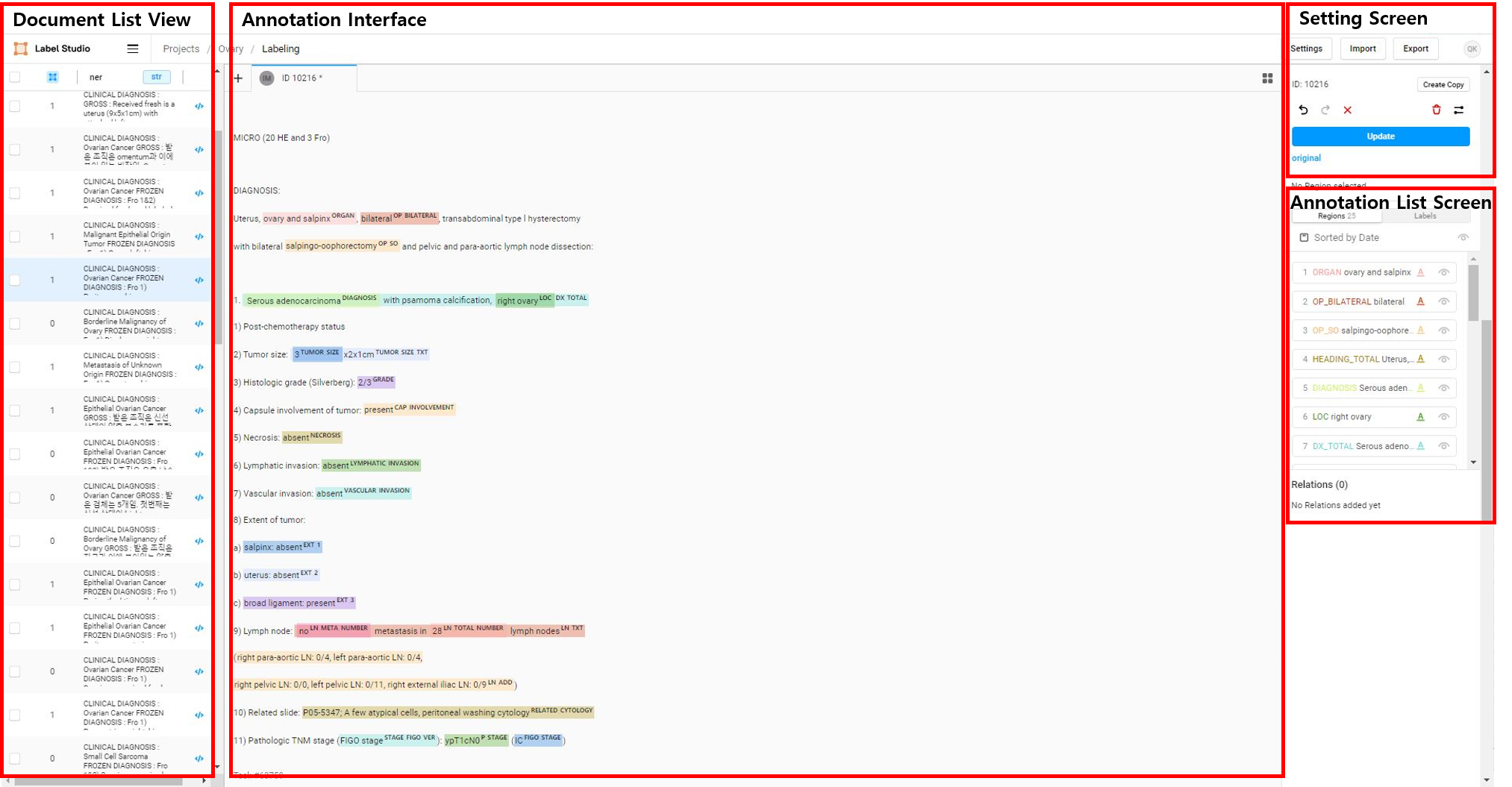

Supplement: S1 Fig — (PNG) [file pone.0318726.s002.png]
