# Supplementary material for: Leveraging natural language processing for efficient information extraction from breast cancer pathology reports: Single-institution study
Source: PLoS One. 2025 Feb 18;20(2):e0318726. doi: 10.1371/journal.pone.0318726 (PMC12005671; doi:10.1371/journal.pone.0318726)
Supplement: Attachment — Submitted filename: Responses to comments.docx [file pone.0318726.s003.docx]

**[Responses to comments]**

**Response to Reviewer #1**

**Reviewer 1:** The study demonstrated that BioBERT, among the three BERT models evaluated, achieved the highest accuracy for extracting information from breast cancer pathology reports, with an average performance of 0.99901. However, there is some room for improvement.

🡪 We appreciate the comprehensive review. We have attempted to revise our manuscript in accordance with the reviewer’s suggestions and comments. Our responses to each of the points raised are provided below.

**Comments:**

**Comment 1**. The paper states, "A total of 13,751 breast cancer surgical pathology reports were collected. Among these, 1,215 pathology reports were used to build annotated data to develop an extraction algorithm for pathology reports," but does not provide a reason why only 1,215 reports were used out of the 13,751 collected.

🡪 Thank you for your detailed comment. As commented by you, we have provided a reason why 1,215 reports were used out of the 13,751 collected in the Methods section of the revised manuscript as follows:

“Our study encompassed a comprehensive dataset of 13,751 breast cancer surgical pathology reports. To ensure a manageable and representative sample for annotation, we employed a random selection process to extract approximately 10% of the reports (1,215 reports in total). Subsequently, we implemented a strategic division of these annotated reports, allocating them to training and test sets in a 7:3 ratio. This methodical approach resulted in a training set comprising 851 reports, while the remaining 364 reports were designated as the test set. This carefully curated dataset served as the foundation for developing and rigorously evaluating our pathology report extraction algorithm.”

**Comment 2**. The paper provides an insufficient background and literature review. A more thorough review of existing methods and their limitations would strengthen the case for using BioBERT and provide a clearer context for the study.

🡪 In response to your comment, we have described the details about the BERT models including BioBERT and ClinicalBERT in the Background section as follows:

“Our primary object was to evaluate the efficacy of various NLP models, specifically BERT-basic, BioBERT, and ClinicalBERT, in accurately extracting information from pathology reports. The selection of these BERT-based models was informed by their specialized pre-training: BioBERT, having been pre-trained on biomedical corpora, is particularly adept at biomedical text analysis, while ClinicalBERT, pre-trained on clinical texts, potentially offers the enhanced performance on clinical documents such as pathology reports. For example, BioBERT has demonstrated exceptional capabilities in biomedical named entity recognition tasks, whereas ClinicalBERT has shown proficiency in extracting clinical concepts.”

**Comment 3**. Figures such as the flowchart and Figure 3 are hard to understand. The flow and content of these figures are not clearly explained, making it difficult for readers to follow the methodology and results.

🡪 Thank you for your appropriate comment. According to your suggestions, we have clearly revised the contents of Figures. In addition, more detailed explanation of the figure's content and flow have been added in Method section. We hope that these changes will help readers better understand the methodology and results of this study.

Please refer to the revised Figures.

Your feedback has been instrumental in enhancing the overall quality and accessibility of our paper, and we are grateful for your constructive criticism.

**Response to Reviewer #2**

**Reviewer 2:** In this paper, the authors developed an information extraction model to extract key information from pathology reports. The authors compared the deep learning models with the regular expression method. I have some comments for this study.

🡪 We appreciate the comprehensive review. We have attempted to revise our manuscript in accordance with the reviewer’s suggestions and comments. Our responses to each of the points raised are provided below.

**Comments:**

**Comment 1**. The innovation of methodology seems weak. What are the differences between this study and others?

🡪 Thank you for your appropriate comment. We agree with your comment that the innovation of methodology seems weak. However, the purpose of this study was not to develop a novel NLP method, but rather to evaluate and select the most optimal model for our specific pathology reports. We utilized existing methodologies, particularly BERT-based models, focusing on adapting and optimizing them for our unique dataset. This approach allowed us to leverage proven techniques while tailoring them to our specific needs.

By comparing multiple BERT-based models (BioBERT, ClinicalBERT, and BERT-basic), we were able to identify the most effective model for our particular use case. This comparative analysis and optimization process is a key differentiator of our study, as it provides valuable insights into the performance of these models in the context of breast cancer pathology reports.

In addition, there are differences in the results between this study and other studies. Previous studies using pre-BERT models mainly focused on extracting information from a limited set of fields in pathology reports, whereas our study demonstrates a key strength in its ability to extract information from a wide range of fields. This comprehensive approach allows for a more accurate analysis of clinicopathological parameters in breast cancer patients.

We hope that this will be accepted.

**Comment 2**. The annotation guideline should be posted in the supplement.

🡪 It is a good comment which needs to be considered. As commented, we have added the annotation guideline (S1 File. Comprehensive annotation guideline for breast cancer surgical pathology reports) and this content has been also described in Methods section.

Please refer to the S1 file.

**Comment 3**. Adding some annotation examples is recommended.

🡪 We appreciate your suggestion to include annotation examples. In response to your recommendation, we have created annotation examples and added them as a supporting information figure (S1 Fig) to our paper. These examples will help readers understand our annotation process more clearly.

Please refer to the S1 Fig.

**Comment 4**. The authors should provide a detailed introduction about how they conduct the question-and-answer task when training IE models.

🡪 According to your suggestion, the following description has been added in the second paragraph of the Data modeling.

“BERT-based models can be fine-tuned based on annotation data to solve specific downstream tasks. In this study, the task of extracting unstructured information was framed as a question-and-answer format, identifying 19 types of entities. To fine-tune for Question and Answer, we used the same BERT architecture used for SQuAD [12], Additionally, we followed the same pre-training process as established methods [13], which uses SQuAD.”

**Comment 5**. The cross-validation, hyperparameters, and hardware should be placed in an experimental setup section.

🡪 Thank you for your feedback. As commented, the information about cross-validation, hyperparameters, and hardware specifications have been moved to the methods section, specifically under a data modeling

**Comment 6**.  Why did the authors use the fuzzy-wuzzy algorithm to compare the performance? Why did the authors not compare the extracted results of these two methods directly?

🡪 Thank you for your appropriate comment regarding our performance evaluation methodology. We would like to clarify that we did not use the fuzzy-wuzzy algorithm for comparing performance. To avoid confusion for readers, we have deleted the fuzzy-wuzzy algorithm and have clarified Named Entity Recognition (NER) for our performance evaluation in Methods and Results sections as follows:

“The performance metrics for the NER (Named Entity Recognition) models were calculated using standard evaluation measures. Precision, recall, and F1-score were computed for each entity type extracted from the pathology reports. Precision measures the proportion of correctly identified entities among all extracted entities, while recall measures the proportion of correctly identified entities among all actual entities in the dataset. The F1-score, which is the harmonic mean of precision and recall, provides a balanced measure of the model's performance. These metrics were calculated for BioBERT model and regular expression to assess their effectiveness in extracting clinical information from the pathology reports in Fig 3.”

**Comment 7**.  The comparison between biobert and regular expression seems inappropriate.

🡪 Thank you for your detailed comment. We agree with your comment that the direct comparison between BioBERT and regular expression seems inappropriate. However, in some respects, the comparison between the two methods may be worthwhile for the following reasons:

To extract information from pathology reports, we initially used the regular expression approach due to its simplicity and low implementation barrier (revised Figure 3). However, as data complexity increases, rule-based systems often required the extensive exception handling, leading to decreased accuracy and increased maintenance challenges. For these reasons, this study aimed to establish a natural language processing model approach that overcomes the limitations of the existing traditional model, regular expressions, by comparing the natural language processing model with existing processed regular expressions.

Our findings may provide valuable insights into the importance of an advanced NLP models approach, compared to traditional regular expressions when extracting information from cancer pathology reports.

We hope that this will be accepted.

**Comment 8**.  As an IE task, precision, recall, F1 score should be reported. Using accuracy only is not enough.

🡪 Thank you for your valuable feedback regarding the performance metrics. As commented, we have reported not only accuracy but also precision, recall, and F1 score in our performance evaluation.

Please refer to the revised Table 2.
