# Supplementary material for: Leveraging natural language processing for efficient information extraction from breast cancer pathology reports: Single-institution study
Source: PLoS One. 2025 Feb 18;20(2):e0318726. doi: 10.1371/journal.pone.0318726 (PMC12005671; doi:10.1371/journal.pone.0318726)
Supplement: Attachment — Submitted filename: Responses_to_comments_auresp_2.docx [file pone.0318726.s004.docx]

**[Responses to comments]**

**Response to Reviewer #2**

**Reviewer 2:** Thanks for considering my comments. All my concerns have been addressed. I think this paper is ready for acceptance.

🡪 Thank you very much for your thorough review and positive feedback. We greatly appreciate your time and effort in evaluating our manuscript. Your constructive comments have helped us improve the quality of our paper significantly. We are grateful for your recommendation for acceptance.

**Response to Reviewer #3**

**Reviewer 3:** The authors addressed most of the reviewers' comments and questions. However, some important information is still missing.

🡪 We appreciate the comprehensive review. We have attempted to revise our manuscript in accordance with the reviewer’s suggestions and comments. Our responses to each of the points raised are provided below.

**Comments:**

**Comment 1**. Reviewer 1, comment 2 ("The paper provides an insufficient background and literature review."): A "Related work" section is still missing. The authors should cite some similar works on information extraction in the biomedical field and highlight the strengths of their work in comparison.

🡪 In response to your comment, we have described the details about previous studies in the Background section as follows:

“Although previous studies have structured pathology reports using NLP, the number of variables that can be extracted is limited.^8-10^ For example, studies that extracted three to four variables, such as pathology grade, lesion location, and treatment method from pathology records or extract only biomarker information through the lymphoma classification tool, or immunopathology information, for a specific purpose have been mainly conducted.^8^”

8. Kim Y, Lee JH, Choi S, et al. Validation of deep learning natural language processing algorithm for keyword extraction from pathology reports in electronic health records. Scientific reports. 2020;10(1):20265.

9. Kefeli J, Berkowitz J, Acitores Cortina JM, Tsang KK, Tatonetti NP. Generalizable and automated classification of TNM stage from pathology reports with external validation. Nature Communications. 2024;15(1):8916.

10. Buckley JM, Coopey SB, Sharko J, et al. The feasibility of using natural language processing to extract clinical information from breast pathology reports. Journal of pathology informatics. 2012;3(1):23.

**Comment 2**. The authors added an annotation example as a supplementary file. They should instead include it in the paper and appropriately comment and explain it.

🡪 It is a good comment which needs to be considered. As commented, we have explained the annotation guideline (S1 File. Comprehensive annotation guideline for breast cancer surgical pathology reports) and example of annotation (S1 Fig. Example of annotated pathology reports for NLP task) this content has been also described in Methods section.

“Annotation guidelines were developed through repeated discussions with the pathologists to obtain standard annotations (S1 File). Following the annotation guideline, annotator worked with pathology reports in a structured interface divided into four main sections: documents list, annotation interface, setting screen, and annotation list (S1 Fig).”

**Comment 3**. The authors added some information on how they conducted the question-and-answer task when training IE models. However, they do so by citing another paper, while they should also add an explanation for those who aren't familiar with that paper.

🡪 According to your suggestion, the following description has been added in the second paragraph of the Data modeling.

“We implemented a question-answering (QA) framework to extract information from pathology reports. This framework converts pathology report fields into questions and their corresponding values into answers, creating a structured approach to information extraction. For instance, when analyzing a pathology report, a field like "Tumor Site" becomes a question that seeks specific location information within the report. Our framework processes various types of clinical information, including tumor characteristics, histological details, and lymph node status. By using the SQuAD (Stanford Question Answering Dataset) architecture adapted for medical contexts, we developed a system that could understand and process medical terminology effectively [15]. Additionally, we followed the same pre-training process as established methods [16], which uses SQuAD. This approach allows the model to analyze pathology reports in a way that mirrors how medical professionals read and interpret these documents.”

**Comment 4**. They should also explain how they evaluate models’ answers. In an IE extraction task, it is important to explain what is considered a false positive/negative if/when the extracted text partially match the annotations.

🡪 Thank you for your appropriate comment regarding our performance evaluation methodology. we have clarified Named Entity Recognition (NER) for our performance evaluation in Methods sections as follows:

“The performance metrics for the NER (Named Entity Recognition) models were calculated using standard evaluation measures. In short, an extraction wass considered a true positive when it successfully captures all essential information from the annotated text, even if the exact boundaries show minor variations. False positives occur when the extracted text either includes irrelevant information or fails to capture critical components of the annotation. Cases where the model fails to extract information or produces incomplete extractions are classified as false negatives.”

**Comment 5**. More generally, it is still unclear how the task is really conducted. The authors should include some examples of questions/answers used to train the models and explain what is given as input to the models during evaluation.

🡪 Thank you for your feedback. As commented, the example about question/answers framework have been added to the methods section as follows:

“For instance, when analyzing a pathology report, a field like "Tumor Site" becomes a question that seeks specific location information within the report.”

**Comment 6**.  Were Bert models fine-tuned without a validation set? Only train/test? Why?

🡪 Thank you for your question about our validation approach. We did not use a separate validation set, due to the small sample size. However, as an alternative, we employed k-fold cross-validation for model evaluation. Through k-fold cross-validation, we were able to effectively assess our model's performance using only train and test sets, as the cross-validation process itself provides robust validation of the model's performance across multiple data splits.

This approach allowed us to maximize the use of our available data while maintaining rigorous evaluation standards. The final performance metrics reported in our study represent the averaged results across all k-fold iterations, ensuring reliable and generalizable results.

**Comment 7**.  What is the metric reported in Table 1? Accuracy or F1? In the data modelling paragraph, the authors talk about F1, while in the Result paragraph they talk about accuracy and the table doesn't specify it.

🡪 Thank you for bringing up this important point about the metrics in Table 1. We have addressed this by clearly specifying both accuracy and F1 scores in the table and ensuring consistency throughout the paper. In the data modeling section, we have discussed F1 scores as they provide a balanced measure of precision and recall. We have also present both accuracy and F1 scores to give a comprehensive view of our models' performance in the Results section. In addition, we have updated Table 1 to clearly label these metrics and maintain consistency with the discussion in both sections.

“Compared to BERT (accuracy: 0.96818, F1 score: 0.96781), it was confirmed that the accuracy of ClinicalBERT (accuracy: 0.97934, F1 score: 0.98092) and BioBERT (accuracy: 0.98609, F1 score: 0.98605) was relatively high, but BioBERT was considered the most suitable.”
